# Supplementary material for: Neuropathic MORC2 mutations perturb GHKL ATPase dimerization dynamics and epigenetic silencing by multiple structural mechanisms
Source: Nat Commun. 2018 Feb 13;9:651. doi: 10.1038/s41467-018-03045-x (PMC5811534; doi:10.1038/s41467-018-03045-x)
Supplement: Supplementary file 1 — Supplementary Information [file 41467_2018_3045_MOESM1_ESM.pdf]

Neuropathic MORC2 mutations perturb GHKL ATPase dimerization dynamics and epigenetic silencing by multiple structural mechanisms

Douse *et al.*

Supplementary Information

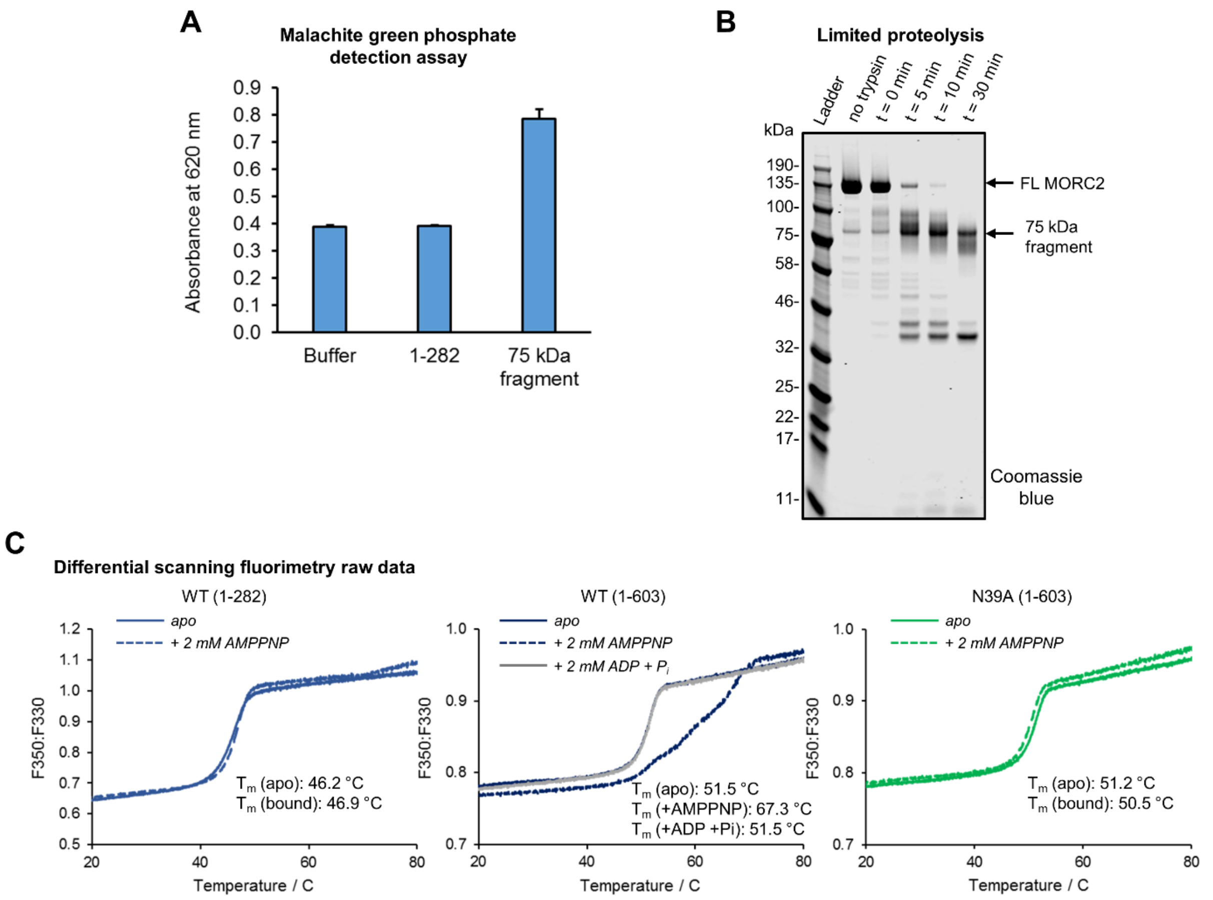

Supplementary Figure 1. Identification, characterization and purification of a MORC2 N-terminal fragment with ATPase activity

(A) Endpoint Malachite green ATPase assay (based on chromogenic detection of evolved phosphate) with different MORC2 constructs. MORC2(1-282) had no detectable activity, while the 75-kDa fragment isolated from degraded full-length material showed evidence of activity. Error bars represent standard deviation between three measurements.

(B) Coomassie-stained SDS-PAGE gel following limited tryptic proteolysis with 10 µg of purified full-length MORC2.

(C) The nano-differential scanning fluorimetry (DSF) thermal unfolding profiles of wild-type MORC2(1-282), (1-603) and N39A MORC2(1-603) in the absence and presence of ligands as marked. See also Fig. 1B.

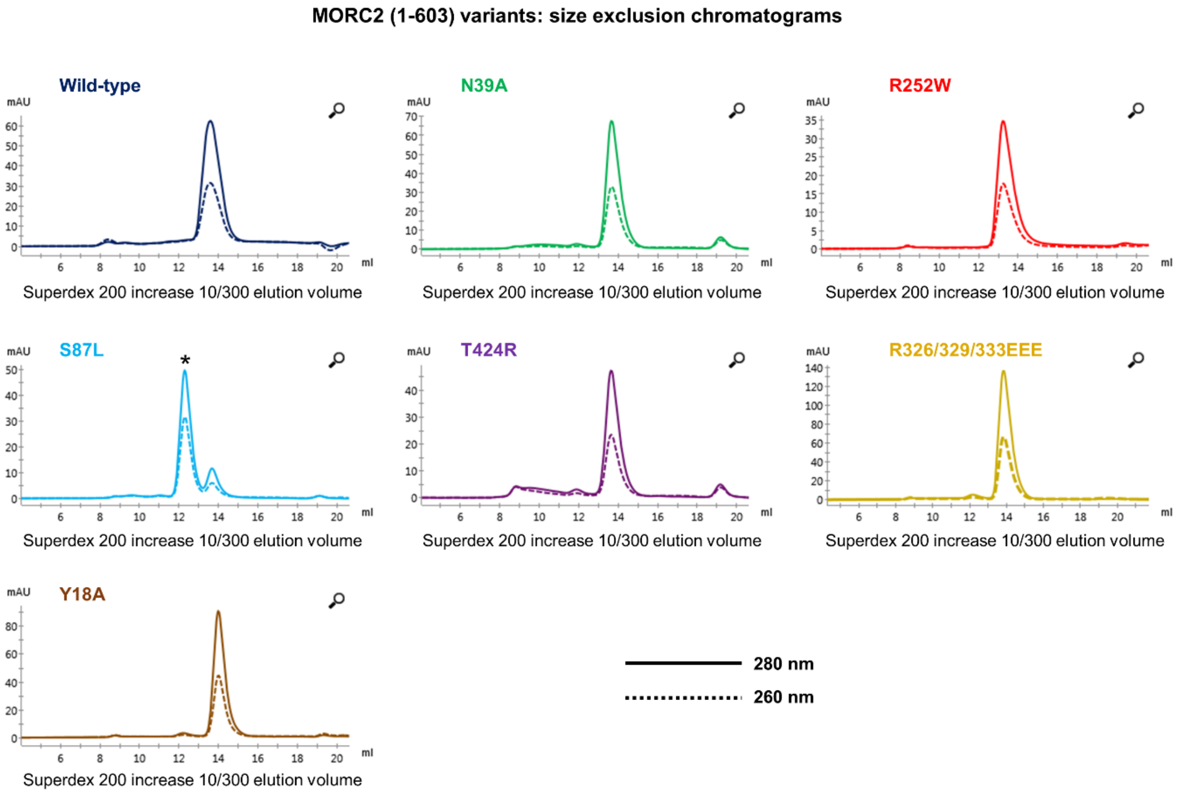

Supplementary Figure 2. Size exclusion chromatograms for all MORC2(1-603) variants reported in this paper. Solid and dashed lines indicate absorbance at 280 nm and 260 nm, respectively. Elution volumes of ~14 mL are consistent with monomeric protein, confirmed for several variants by MALS (see also Figs. 1C, 2F and 5C). In the S87L trace, the asterisk marks the species that elutes early which we assign as a nucleotide-bound dimer. Chromatography was performed with a S200 increase (10/300) column (GE Healthcare).

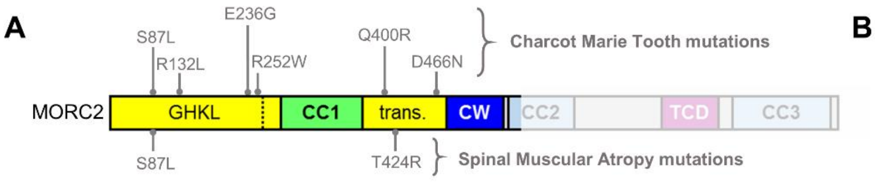

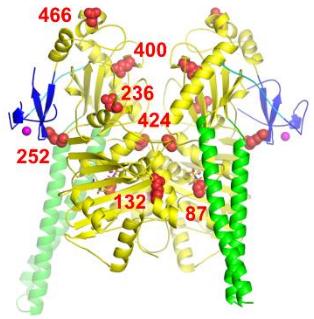

C

Zn energy scan	Energy (keV)		f`		f``	
	Peak	*Inflection*	*Peak*	*Inflection*	*Peak*	*Inflection*
Crystal 14	9.72827	9.66393	-4.59	-9.57	4.56	2.99
Crystal 17	9.68139	9.66393	-6.19	-9.36	4.69	2.84

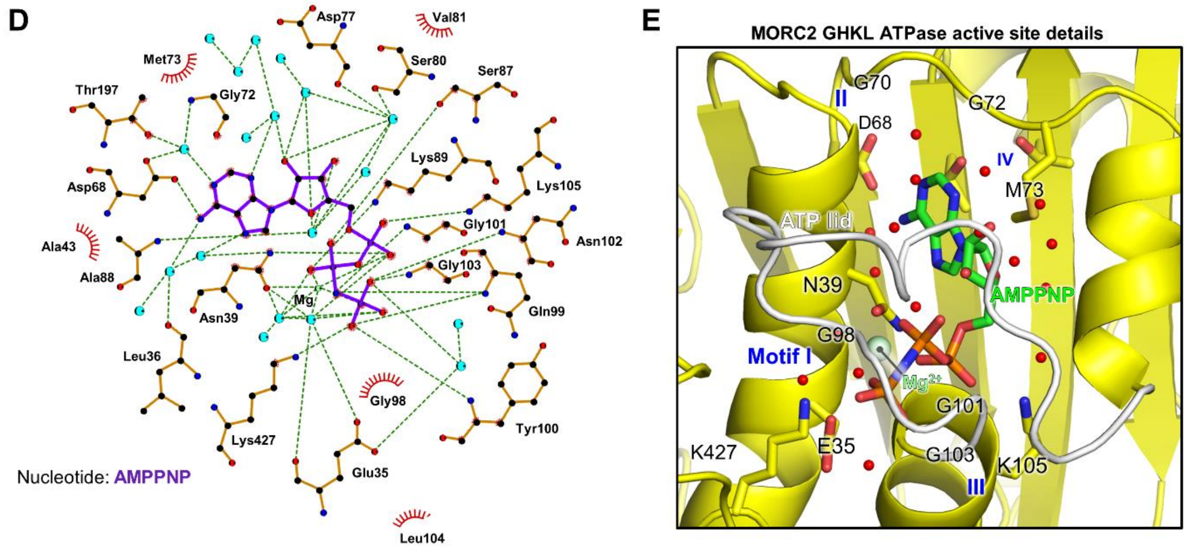

Supplementary Figure 3. Supporting details related to the crystal structure of the MORC2(1-603)-AMPPNP homodimeric complex.

(A and B) Positions of neuropathic (CMT- and SMA-associated) point mutations on the MORC2 domain structure (A), and 3D structure (B). The side chains of mutated residues are shown as red spheres.

(C) X-ray fluorescence spectra have absorption peaks at energies near the theoretical zinc *K* absorption edge (inflection point 9.6586 keV), indicating that zinc is bound in two MORC2(1-603)-AMPPNP crystals tested (Crystals 14 and 17).

(D and E) Details of the GHKL-type ATPase active site of MORC2. The recognition of the non-hydrolysable ATP analogue AMPPNP is shown schematically (D), and in 3D to illustrate the four motifs specific to the GHKL family (E). Water molecules are shown as blue circles in (D) and as red spheres in (E).

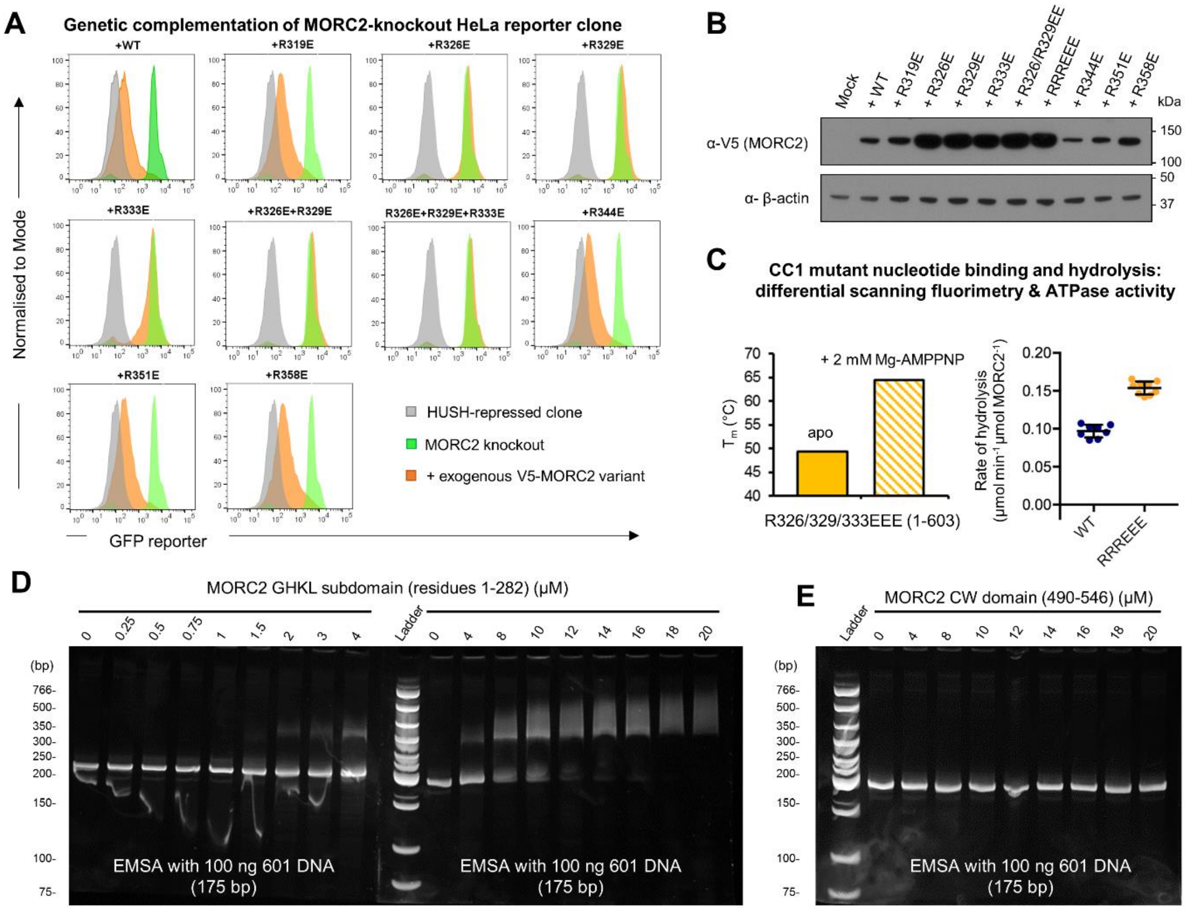

Supplementary Figure 4. Functional importance and DNA binding of CC1 and other MORC2 domains.

(A and B) FACS plots of genetic complementation assays to assess HUSH-dependent silencing of CC1 charge reversal mutants. Shown are the data from Day 12 post-transduction: in grey is the GFP reporter fluorescence of the repressed clone; in green is the MORC2 knockout; in orange is the MORC2 knockout transduced with exogenous MORC2 variants (A). The lentiviral vector used expresses mCherry from an internal ribosome entry site (IRES), enabling control of viral titre by mCherry fluorescence measurement. A Western blot of cell lysates (B) shows that despite using the same MOI the inactive MORC2 variants were expressed at higher concentrations than WT.

(C) The triple CC1 mutant R326/329/333EEE is folded and an active ATPase according to DSF (left) and ATPase (right) assays. Quoted T_m values are an average of two technical replicates; note that the deviation between measurements was <0.2 °C in all cases. For the ATPase assay, error bars represent standard deviation between measurements; n=8 (WT), n=9 (RRREEE). The WT data are as shown in Fig. 1D.

(D and E) EMSAs with 100 ng 601 DNA in the presence of increasing concentrations of MORC2 GHKL domain (D) or CW domain (E). Gels were post-stained for DNA with SYBR Gold. Note that in (D), the samples were run in two different gels, stained and scanned next to each other. The 0 μM and 4 μM protein samples were run in both gels as an internal control.

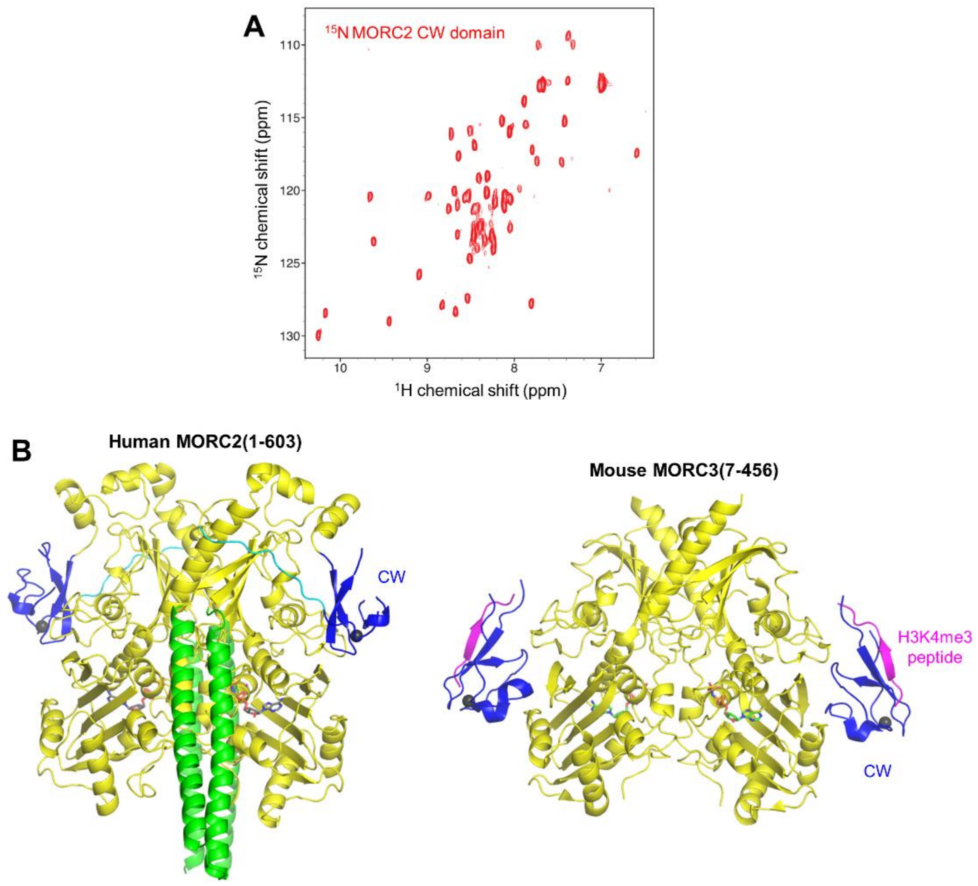

Supplementary Figure 5. Supporting details concerning the structure and function of the MORC2 CW domain.

(A) The MORC2 CW domain does not require the ATPase domain for folding. The ^{1}H, ^{15}N-HSQC spectrum of isolated ^{15}N-labelled MORC2 CW domain (residues 490-546) at pH 7.5 and 298 K shows amide chemical shift dispersion consistent with a folded domain.

(B) Comparison of the overall human MORC2 and mouse MORC3 ATPase-CW structures (cartoon representation, with nucleotides shown in stick representation and metal ions shown as space-filling spheres) shows the different position of the MORC2 CW domain as compared to MORC3, relative to the ATPase module. Domains making up the ATPase modules are in yellow, CW domains in blue, CC1 in green (MORC2 only), H3K4me3 peptide in pink (MORC3 only).

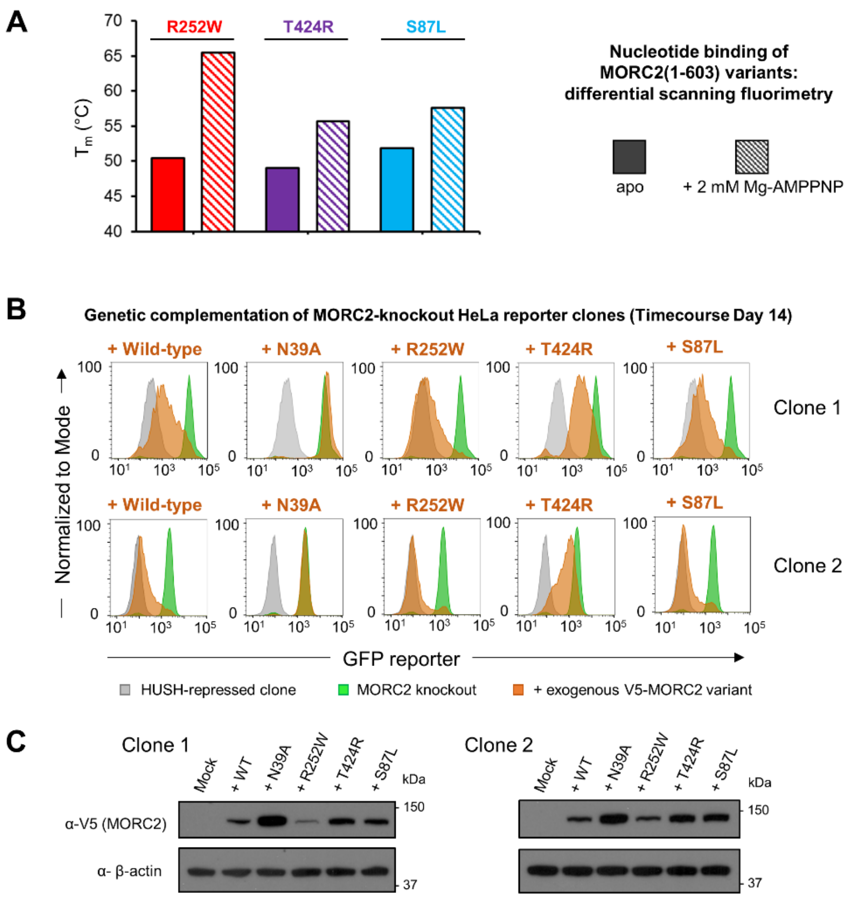

Supplementary Figure 6. Nucleotide binding and HUSH-dependent silencing activities of neuropathic variants of MORC2.

(A) Summary of nano-differential scanning fluorimetry (DSF) data with different MORC2(1-603) disease variants in the absence (solid bar) and presence (striped bar) of 2 mM Mg-AMPPNP. The neuropathic mutations studied here do not cause unfolding of the ATPase module and all variants showed substantial thermal stabilization in the presence of AMPPNP. Quoted T_m values are an average of at least two replicates; note that the deviation between measurements was <0.2 °C in all cases.

(B) Example FACS plots from the timecourse complementation experiments in two different clones, shown in Fig. 5b. Shown are the data from Day 14 post-transduction: the GFP reporter fluorescence of the HUSH-repressed clone is in grey; the MORC2 knockout is in green; the MORC2 knockout transduced with exogenous MORC2 variants is in orange.

(C) Western blot validation of expression of the exogenous MORC2 variants in each MORC-knockout reporter clone.

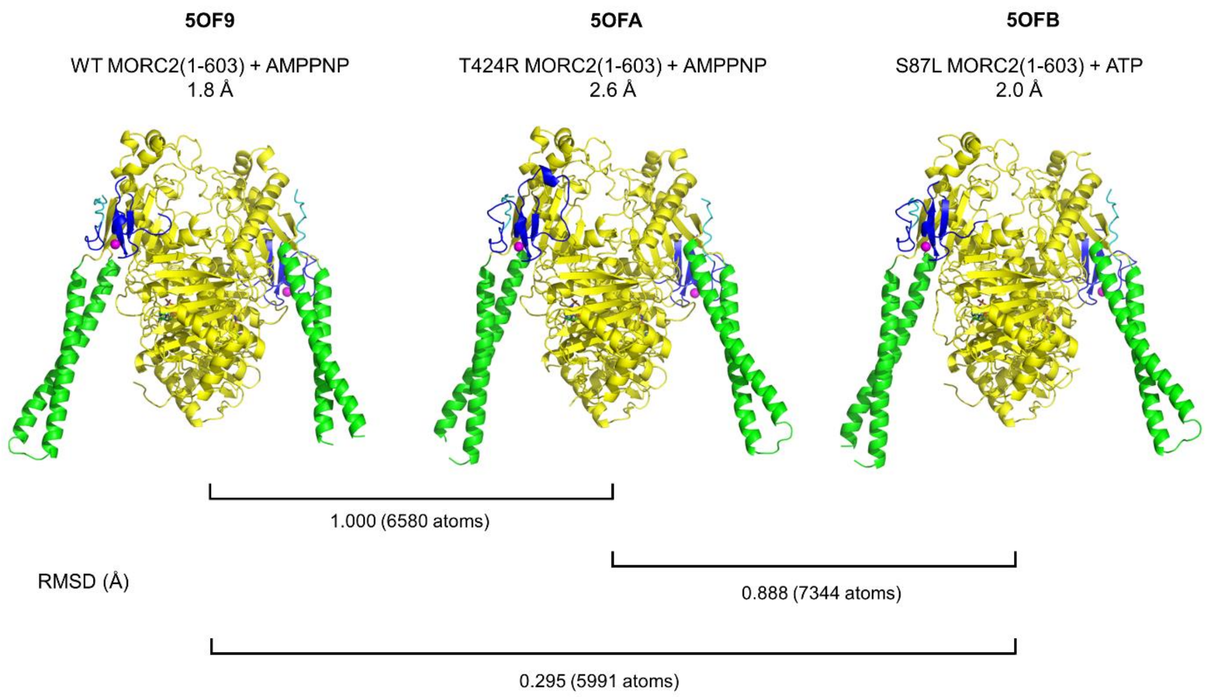

Supplementary Figure 7. Comparison of the overall structures of MORC2(1-603) variants reported in this paper. Root mean square deviations (RMSDs) for all atoms in each structure pair are listed.

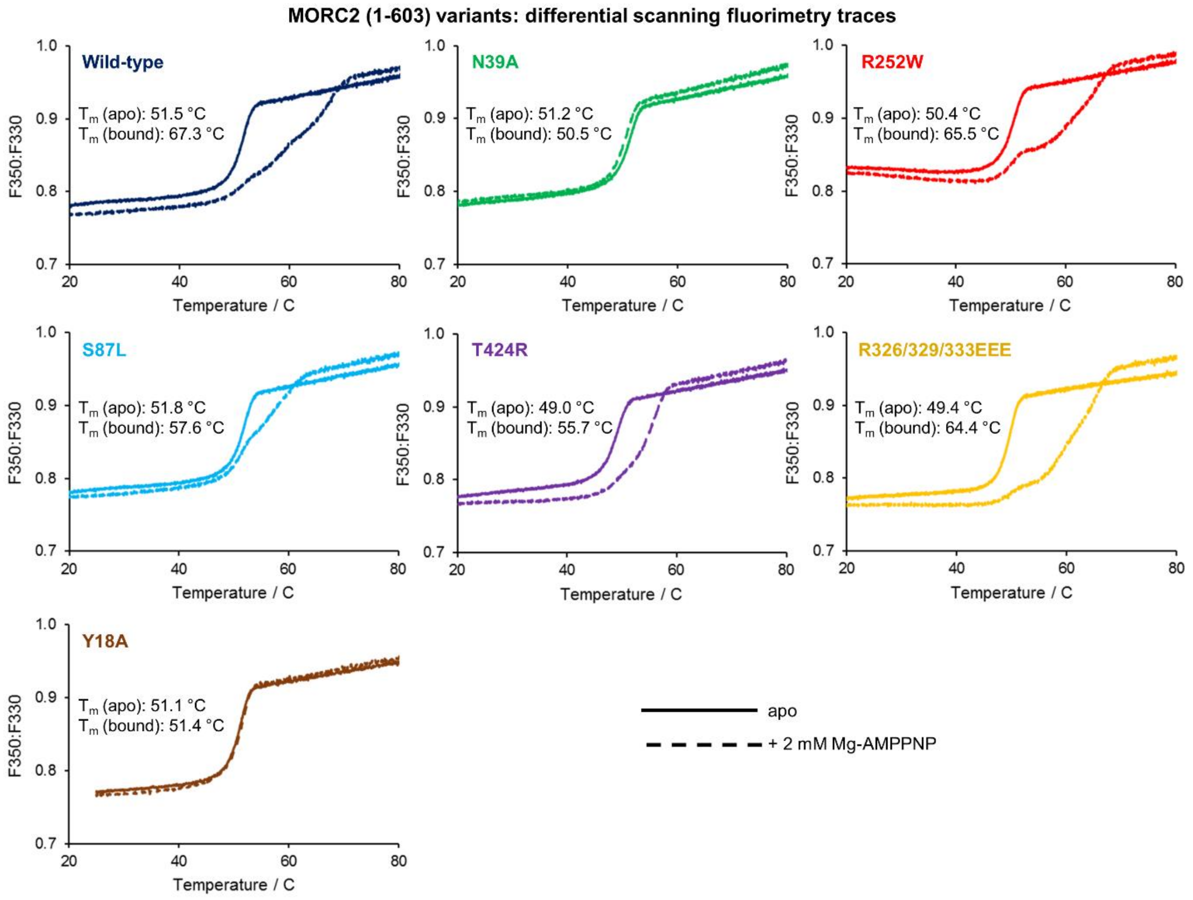

Supplementary Figure 8. The nano-differential scanning fluorimetry (DSF) thermal unfolding profiles of all MORC2(1-603) variants reported in this paper (5 µM), in the absence (solid line) and presence (dashed line) of 2 mM Mg-AMPPNP. The fitted T_m values are shown.

A

B

C

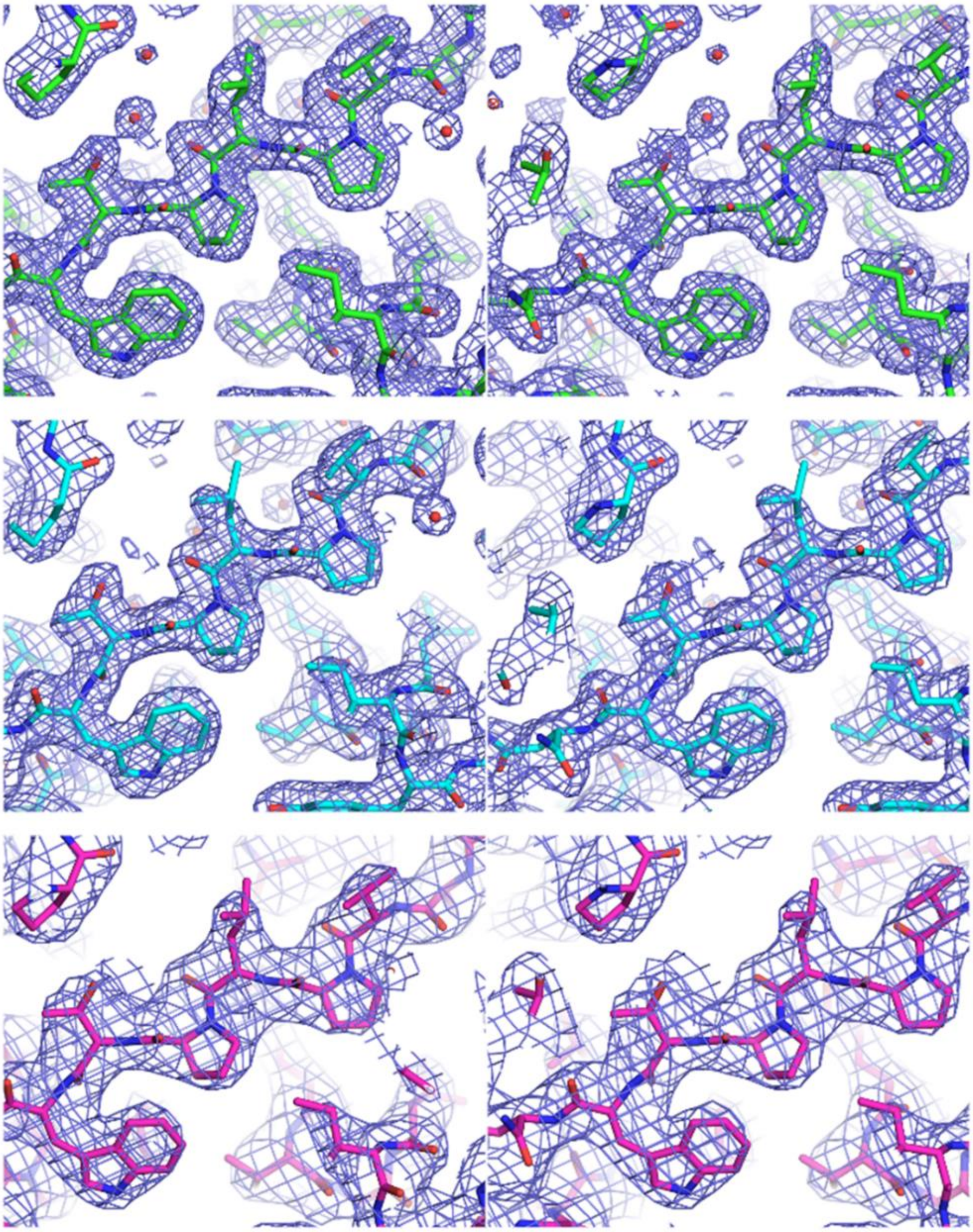

Supplementary Figure 9. Stereo images of $2F_{obs}$-F_{calc} electron density maps for portions of the three crystal structures reported in this paper. Maps were calculated using the refined coordinates of wild-type (A, green), S87L (B, cyan) and T424R (C, purple) MORC2 variants. All three maps are contoured at 1σ.

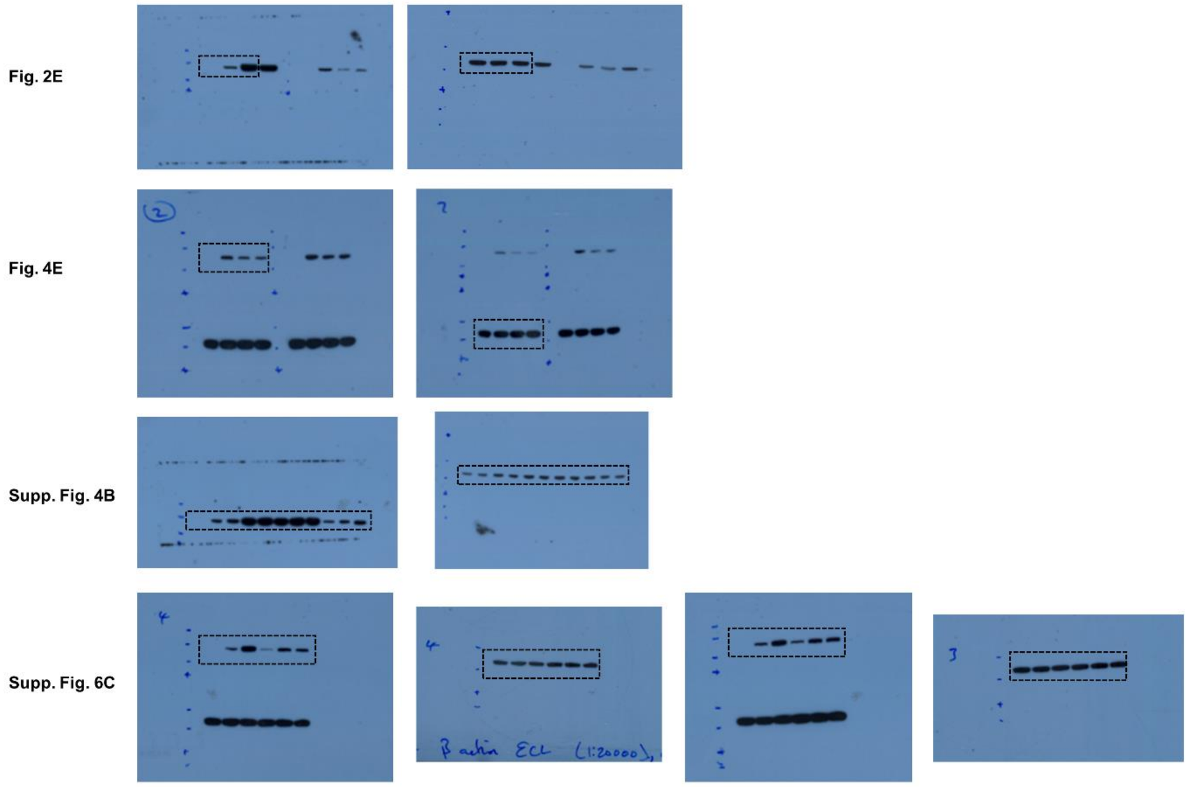

Supplementary Figure 10. Uncropped Western blot images.

Supplementary Table 1. Oligonucleotide sequences

	Sequence (5' to 3')
MORC2_N_f	ATGGCTTTCACAAATTACAGCAGTCT
MORC2_C_r	TCAGTCCCCCTTGGTGATGAG
MORC2-603_r	TCAAGTGGAAGGTCTGGTGGT
N39A_f	ACTGGTTGATGCTGCAAGAGATGCTGATGCCACCAG
N39A_r	CATCTCTTGCAGCATCAACCAGTTCAGCAAGGGCAC
S87L_f	GTTTGGGAAGTTGGCCAAGCGAACACCTGAGTCTAC
S87L_r	TTCGCTTGGCCAACTTCCCAAACTGGATCACACTG
T424R_f	CCTGGAGCCTAGGCACAACAAACAGGACTTTGC
T424R_r	GTTTGTTGTGCCTAGGCTCCAGGACCAGGTAGG
Y18A_f	GCTAACCTTTGAAGCTCTGCACACAAATTCAACCACTC
Y18A_r	TTGTGTGCAGAGCTTCAAAGGTTAGCTGAGCTCG
R326329333EEE_f	GAGACCTCACGGAGGACTCCGAGGTGATGTTGGAACAGGTCCAGAACAGAGCCATC
R326329333EEE_r	CTGGACCTGTTCCAACATCACCTCGGAGTCCTCCGTGAGGTCTCCACCTAGGCG
R266A_f	CAGACCAAGGCGCTCTCCTGCTGCCTGTACAAGCCCA
R266A_r	CAGGAGAGCGCCTTGGTCTGCACCTTGTGCCCATG
R319E_f	GACATTAGAAGTAGAGCTAGGTGGAGACCTC
R319E_r	GAGGTCTCCACCTAGCTCTACTTCTAATGTC
R326E_f	GGTGGAGACCTCACGGAGGACTCCAGGGTGATG
R326E_r	CATCACCCTGGAGTCCTCCGTGAGGTCTCCACC
R329E_f	CTCACGCGGGACTCCGAGGTGATGTTGCGACAG
R329E_r	CTGTCGCAACATCACCTCGGAGTCCCGCGTGAG
R333E_f	TCCAGGGTGATGTTGGAGCAGGTCCAGAACAGA
R333E_r	TCTGTTCTGGACCTGCTCCAACATCACCCTGGA
R326329EE_f	GGTGGAGACCTCACGGAGGACTCCGAGGTGATGTTGCGACAG
R326329EE_r	CTGTCGCAACATCACCTCGGAGTCCTCCGTGAGGTCTCCACC
R344E_f	GCCATCACTCTGCGCGAAGAAGCCGATGTCAA
R344E_r	TTGACATCGGCTTCTTCGCGCAGAGTGATGGC
R351E_f	GCCGATGTCAAGAAGGAGATCAAGGAGGCCAAG
R351E_r	CTTGGCCTCCTTGATCTCCTTCTTGACATCGGC
R358E_f	AAGGAGGCCAAGCAGGAAGCACTTAAAGAACCT
R358E_r	AGGTTCTTTAAGTGCTTCCTGCTTGGCCTCCTT
**Sequencing**	
pOET_f	CAAACTAATATCACAAACTGGAAATGTCTATC
pOET_r	TACAACAATTGTCTGTAAATCAACAACGC
SFFV_f	TGCTTCTCGCTTCTATTCG
WPRE_r	CCACATAGCGTAAAAGGAGC
M2-seq1	GATGAAGTGATAGTCCCACTGC
M2-seq2	GCGAGCACTTAAAGAACCTAAGG
M2-seq3	GCAGGAGAAGCTGGAGGCCCTTC
MORC2seq579	GGACAGCGGAACATTGGTG
MORC2seq1236	GGTTGTTGATGTGCCCTACC
MORC2seq1896	GCCAACTCCTAGACCAGCCA
